# Compositional Shifts in Microbial Diversity under Traditional Banana Cropping Systems of Sub-Saharan Africa

**DOI:** 10.3390/biology11050756

**Published:** 2022-05-16

**Authors:** Manoj Kaushal, John Baptist Tumuhairwe, Jacob Kaingo, Malingumu Richard, Florence Nakamanya, Godfrey Taulya, Danny Coyne

**Affiliations:** 1 International Institute of Tropical Agriculture (IITA), Mikocheni B, Dar es Salaam P.O. Box 34441, Tanzania; jacobkaingo@gmail.com; 2 College of Agriculture and Environmental Sciences, Makerere University, Kampala P.O. Box 7062, Uganda; jbtumuhairwe@gmail.com (J.B.T.); g.taulya@cgiar.org (G.T.); 3 Faculty of Agriculture and Environmental Sciences, Muni University, Arua P.O. Box 725, Uganda; malingrich@gmail.com; 4 International Institute of Tropical Agriculture (IITA), Kampala P.O. Box 7878, Uganda; florencekateregga@gmail.com; 5 International Institute of Tropical Agriculture (IITA), Nairobi P.O. Box 30772-00100, Kenya; d.coyne@cgiar.org

**Keywords:** cropping systems, metagenomics, microbial diversity, enzymes, sub–Saharan Africa

## Abstract

**Simple Summary:**

In soil, the connection between microbial diversity and plant health is vital in terms of achieving the food security. Here, we suggested the study on soil microbial diversity in diverse cropping systems of banana adopted and followed by small holder farmers over the years. We tracked down the bacterial and fungal diversity in mono cropping and intercropping systems using advanced molecular techniques. Our outcomes likewise uncovered that the impact of cropping systems on bacterial and fungal increments in plant roots and rhizosphere soil. Hence, safeguarding of soil microbial diversity is profoundly significant taking into consideration of the contributions for plant buildups and rhizodeposits into the soil.

**Abstract:**

Improvements in the crop productivity, soil health, and sustainable intensification should be premised on the better understanding of interactions between the cropping systems and soil microbial diversity. In this study, we assessed variations in the microbial communities across the traditional banana-based cropping systems of contrasting monocrop vigor (vigorous or V vs. non-vigorous or NV) and the cropping system (monocrop or MC vs. intercropped or IC) using 16S rDNA (V3–V4) and ITS2 amplicon deep sequencing via Illumina platform. Sequencing results of the bacterial and fungal communities showed high variability among MC and V cropping systems. The abundances of Proteobacteria, Firmicutes, and Actinobacteria were significantly higher in NV (non-vigorous) and V (vigorous) cropping systems; and the abundances of Proteobacteria, Firmicutes, Actinobacteria, and Bacteroidetes in the MC (monocropping) than IC (intercropping). There were high relative abundances of *Pseudomonas* (6.1–37.43%), *Bacillus* (4.5–20.4%), *Rhizobium* (1.4–6.5%), and *Devosia* (1.5–6.7%) in the cropping systems. The dominant family of fungal class Incertae_sedis was Mortierellales, which accounted for 8.79–41.12% of total taxa. This result indicated that the cropping systems are vital for supporting the dynamic microbial diversity specifically beneficial for bacterial communities that helps in promoting synergistic plant-soil interactions and total productivity under resource poor conditions of smallholder farmers in sub-Saharan Africa (SSA).

## 1. Introduction

The plant performance highly depends on the particular plant-soil-biological interaction in the rhizosphere, i.e., the region to immediate surroundings of the roots. This rhizospheric black box hosts most of the complex and dynamic plant-microbial crosstalk’s responsible for plant and soil health improvements. Microbes play a crucial role in various ecosystem functioning such as nutrient cycling, soil structure stabilization, pollutant removal, decomposition of organic matter, and buffering soil pH [1,2]. These sensitive biological indicators benefit plants with many positive interactions including mycorrhization, induced systemic resistance, and pathogen suppression through enhanced defense mechanisms [3,4,5]. Being vital participants in soil, bacterial and numerous fungal species are generally in the front to ensure overall sustainable productivity and quality of the agricultural crops. Therefore, understanding the diversity of soil microbes is a prerequisite to unravelling soil functionality and crop response to the soil management practices. Integrated management practices and inputs influences the endurance and composition of soil microbiota. Thus, soil microbial communities have also attracted wide attention in various crop production systems [6,7]. Increased demand of organic agriculture products has triggered interest of the scientific community towards farming systems comparison on various attributes of soil and plant health. Different cropping systems such as intercropping, mixed cropping, and crop-rotations are common practices deemed necessary to sustain productivity and feed the future. However, intercropping and mixed cropping systems do not always translate into enhanced sustainability of soil health indices and crop productivity. It was illustrated that cereal and legume intercropping systems had lower biomass and accumulation of nitrate compared to the respective monocropping systems [8]. Therefore, comparative studies of soil microbial communities and activity under different cropping systems should be used to scrutinize and develop strategies for improved sustainable agricultural production.

Bananas are a staple food crop and serve as an important component for livelihood of smallholder farmers in the great lake’s region of sub-Saharan Africa (SSA) including Uganda and Tanzania. Tanzania and Uganda together account for 50% of the total banana production in Africa with an annual crop value of USD 4.3 billion. Smallholder cropping systems in SSA are characterized by limited use of external soil fertility inputs yet continuously utilizing the land. This significantly reduces soil microbial diversity cascading to low soil fertility and crop yields, ultimately increasing food insecurity in the region. Bananas in the region are mostly grown either as monocrop or intercropped with coffee and beans. Intercropping bananas with other crops have obvious advantages for yield stability, water and nutrients use, and diminishing impacts of pathogens and diseases in the field apart from the added economic benefits [9,10]. Furthermore, microbial networks have explicit inferring that intercropping and mixed cropping systems support more extravagant and more different microbiomes than monocropping systems [11]. This also helps in improving crop and soil quality by maximizing utility of limited resources and reduced dependence on external farm inputs. Intercropping systems also enhance various soil-microbe bound mechanisms such as nitrogen fixation and mobilization of phosphorus in the rhizosphere [12]. Various crop studies were consistent only on soil fertility, crop yield and quality, and relationships with underground and aboveground microbial diversity, but the results for the comparison of microbial diversity in different cropping systems are still lacking [13,14]. Additionally, studies on microbial diversities have focused on diverse crops ranging from wheat and maize to high return crops such as vegetables [15,16,17]. However, no reports have been published for the comparison of microbial community behaviors in diverse banana farming systems that are common among smallholder farming communities in SSA. Here, we targeted to unravel microbial diversity occurring between two commonly adopted cropping systems of mono (MC) and intercropping (IC) with coffee/beans cropping systems in Uganda and Tanzania. We also assessed microbial diversity of a crop vigorous (V) and non-vigorous (NV) mono banana cropping systems of smallholder farmers in Uganda and Tanzania. For both cropping systems, we explored changes in microbial species from bulk soil to rhizosphere and plant roots.

## 2. Materials and Methods

### 2.1. Study Area and Sample Collection

**Figure 1:**
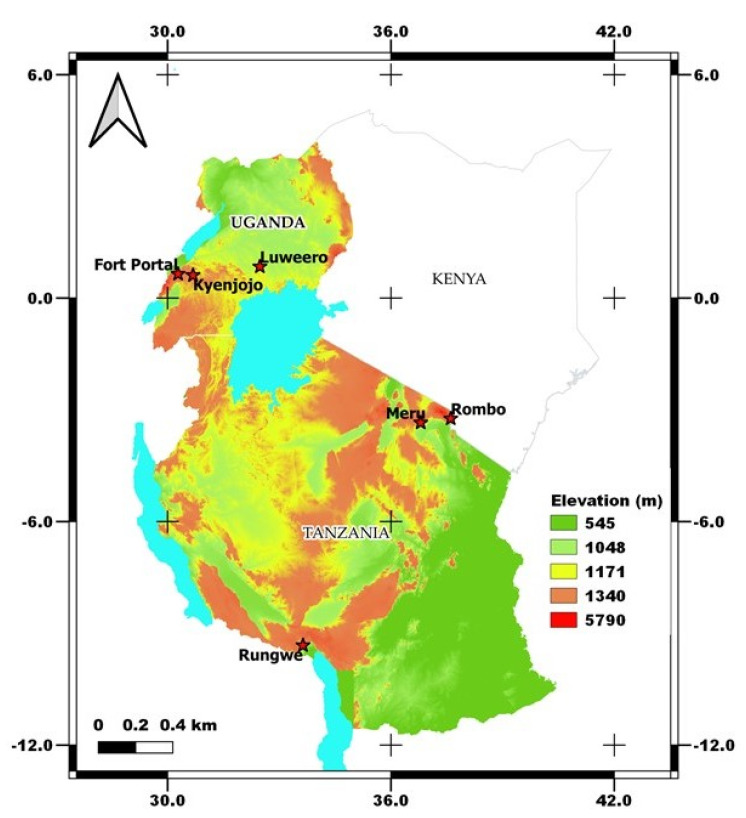
Sampling locations of traditional banana farming systems in Tanzania and Uganda under varied agroclimatic zones. The map was generated in QGIS software (version 3.16-Hannover) using the 30 m resolution Shuttle Radar Topography Mission (SRTM) Digital Elevation Model (DEM).

Smallholder farmers’ banana fields with cooking-type banana cultivars (Sukali Ndizi) were targeted for sampling from two different countries viz. Tanzania and Uganda. We focused on the traditional cropping systems in bananas viz. monocropping (MC) and intercropping (IC) with coffee/beans and fields that were mono banana vigorous (V) vs. those that were non-vigorous (NV) with the same management practices (Supplementary Figure S1). Vigorous (V) cropping systems defines the field with heavy foliage in banana plants that produces large, firm, and smooth fruiting. On the other hand, non-vigorous (NV) cropping systems includes the banana plants with very less foliage and hard to show fruiting (Supplementary Figure S1). In each country, three different banana growing zones were identified which represents the major banana growing regions of the country. The zones in Tanzania are zone 1- Meru, zone 2- Rombo, and zone 3- Rungwe, and in Uganda are zone 1- Fort Portal, zone 2- Luweero, and zone 3- Kyenjojo (Figure 1). These zones in Tanzania can be distinguished with warm wet zone, warm dry, and cold wet zones, however all the zones in Uganda are covered by warm and dry climate.

At each location we targeted 8–10 different smallholder farmer fields that were under the same management practices. Different plants of similar phenological stage were sampled from each field selected in a randomized manner. For sampling of bulk soil, rhizosphere soil, and roots, three random locations around a single plant were targeted. Bulk and rhizosphere soil samples were collected from 15 to 30 cm depth and root samples were collected at similar depth after removal of attached soil. For microbial diversity analysis, samples were pooled for each cropping system representing smallholder banana growing farmers’ fields. Samples were immediately kept in polythene sampling bags and carried to the laboratory in an ice-cold box. Samples were further kept at 4 °C in the laboratory until processed. All procedures were carried out according to the institutional guidelines and regulations (International Institute of Tropical Agriculture). Prior permissions were obtained from each farmer for soil and root sampling.

### 2.2. Soil Physico-Chemical Analysis and Enzymatic Activities

Soil samples were analyzed for soil orders and different physio-chemical parameters, including texture, pH, soil organic carbon (SOC), total nitrogen (TN), available phosphorus (P), Sulphur (S), potassium (K), calcium (Ca), magnesium (Mg), and sodium (Na). The pH was measured with a pH meter/potentiometer in a soil: water ratio of 1:2.5. Soil OC content was determined by Walkley and Black method using the potassium dichromate. The TN was determined by Kjeldahl method, and available P was determined by NaHCO_3_ extraction molybdenum-antimony colorimetry [18,19].

Activities of invertase, urease, catalase, and phosphatase in the soil samples from all locations were measured. Activities of invertase, urease, catalase, and phosphatase were determined by 3,5-dinitrosalicylic acid colorimetry, indophenol blue colorimetry, KMnO_4_ titration, and disodium phenyl phosphate colorimetry methods, respectively [20].

### 2.3. Genomic DNA Extraction and Amplicon Generation

DNA was extracted using the Plant and Soil DNA Isolation Kit (Zymo Research Corp., Irvine, CA, USA). DNA concentration and purity were assessed on 1% agarose gels. 16S rRNA (16SV4/16SV3/16SV3-V4/16SV4-V5) and ITS (ITS1/ITS2, Arc V4) genes of distinct regions were amplified used specific primer with the barcode. All PCR reactions were carried out with Phusion High-Fidelity PCR Master Mix (New England Biolabs, Ipswich, MA, USA). Samples with bright main strip between 400 bp and 450 bp were chosen for further experiments. PCR products were mixed at equal density ratios. The mixed PCR products were purified with Qiagen Gel Extraction Kit (Qiagen, Germany). The libraries were generated with NEBNext UltraTM DNA Library Prep Kit for Illumina, quantified via Qubit and Q-PCR, and were analyzed by Illumina platform.

### 2.4. Sequencing Data Processing

The whole metagenome library preparation and sequencing process was done at NovogeneAIT Genomics Pte Ltd., Tianjin, China using NovaSeq 6000 Library Preparation Kit (TruSeq Nano DNA HT). Paired-end reads was assigned to samples based on their unique barcodes and truncated by cutting off the barcode and primer sequences followed by merging using FLASH (V1.2.7, http://ccb.jhu.edu/software/FLASH/ accessed on 1 November 2011) [21]. Quality filtering on the raw tags was performed to obtain high-quality clean tags according to the QIIME [22]. The tags were compared with the reference database algorithm to detect and remove chimera sequences to obtain the effective tags.

### 2.5. OTU Clustering and Taxonomic Annotation

Sequences analyses were performed by Uparse software [23] using all the effective tags. Sequences with ≥97% similarity were assigned to the same operational taxonomic units (OTUs). Representative sequence for each OTU was screened for further annotation and Mothur software was performed against the SSUrRNA database of SILVA Database for species annotation at each taxonomic rank (Threshold: 0.8~1) [24]. To obtain the phylogenetic relationship of all OTUs representative sequences, the MUSCLE (multiple sequence comparison by log-expectation) was used to rapidly compare the multiple sequences. OTUs abundance information was normalized using a standard of sequence number corresponding to the sample with the least sequences. Subsequent analysis of alpha diversity and beta diversity were performed based on this normalized output data.

### 2.6. Data Analysis

Alpha diversity was applied to the analysis of microbial diversity within the sample that can reflect the richness and diversity of microbial communities in each sample. OTUs generated at 97% sequence identity were considered to be homologous in species calculated using QIIME software and displayed using the qiimer package in R-software [25]. Beta diversity analysis was used to evaluate the differences of samples in species complexity and calculated by QIIME software. For the assessment of variations in microbial community, the sequences were transformed to relative abundance and beta-dispersion analysis of pairwise Bray–Curtis and unweighted uniFrac similarities were done. To visualize significant results, we explored the dissimilarities based on the distance of the respective samples of cropping systems and plotted in a boxplot. Hierarchical clustering analysis preceded by principal coordinate analysis (PCoA) was used to visualize differences in bacterial and fungal community composition among samples. Unweighted pair-group method with arithmetic (UPGMA) means hierarchical clustering algorithm using average linkage was employed to interpret the distance matrix. To test the effect of sampling site and sample types on bacterial and fungal community composition, we used a distance-based redundancy analysis (db-RDA) performed with forward and backward model selection in the vegan package version 2.5-4. Anosim (analysis of similarities), MRPP (multi-response permutation procedures), and Adonis were performed to determine or compare significant differences of community structure between groups and within groups. We performed a PICRUSt analysis to obtain a predictive functional profile of the bacterial and fungal communities. PICRUSt requires OTU abundances mapped to Greengenes OTU IDs as input for prediction of corresponding metagenomes. The predicted gene families and pathways were compared to those from metagenome sequencing in terms of their KEGG annotations [26,27,28].

## 3. Results

### 3.1. Physico-Chemical Parameters of Study Sites

**Figure 2:**
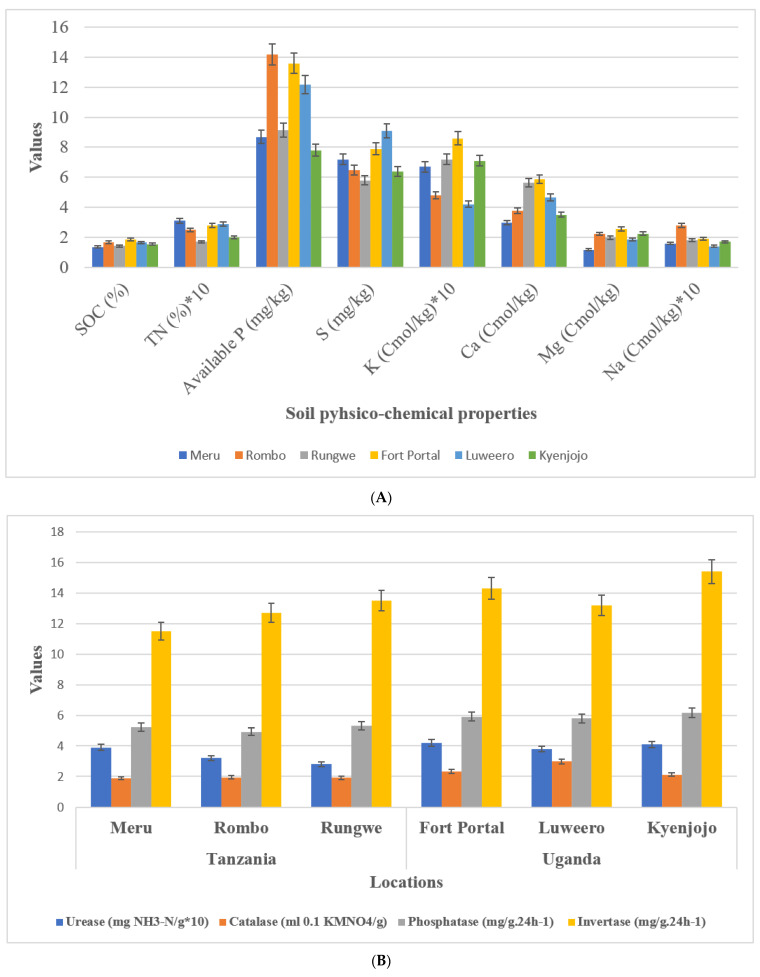
Soil physico-chemical analysis and enzymatic activities. (**A**) Soil chemical properties (**B**) Soil enzymatic activities.

Geographical and metrological parameters of the sampling locations are given in Table 1. Soil texture at sampling locations in both countries was clay loam. Average soil pH in Uganda was 6.8 which was comparatively lower than samples in Tanzania with an average of 7.4. Soil nutrient analyses showed no difference in TN, available P, S, K, Ca, Mg, and Na between Tanzanian and Ugandan soils (Figure 2A). Interestingly, however, SOC content showed a reduction in Tanzanian soils (1.35–1.67) compared to Ugandan soils (1.54–1.87). Three experimental field sites of Tanzania have different soil types of loamy sand (Meru), sand (Rungwe), and sandy loam (Rombo), and Uganda have different soil types of sandy clay loam (Fort portal and Luweero) and sandy clay (Kyenjojo) orders, respectively.

### 3.2. Enzymatic Activities in Study Sites

Soil enzymes including phosphatase, invertase, urease, and catalase of both countries were compared. Among them, the activity of invertase and catalase in Tanzania were lower than those in Uganda. However, there was no difference in urease and phosphatase activity among the locations of the two countries (Figure 2B).

### 3.3. Species Richness of Microbial Communities in Cropping Systems

**Figure 3:**
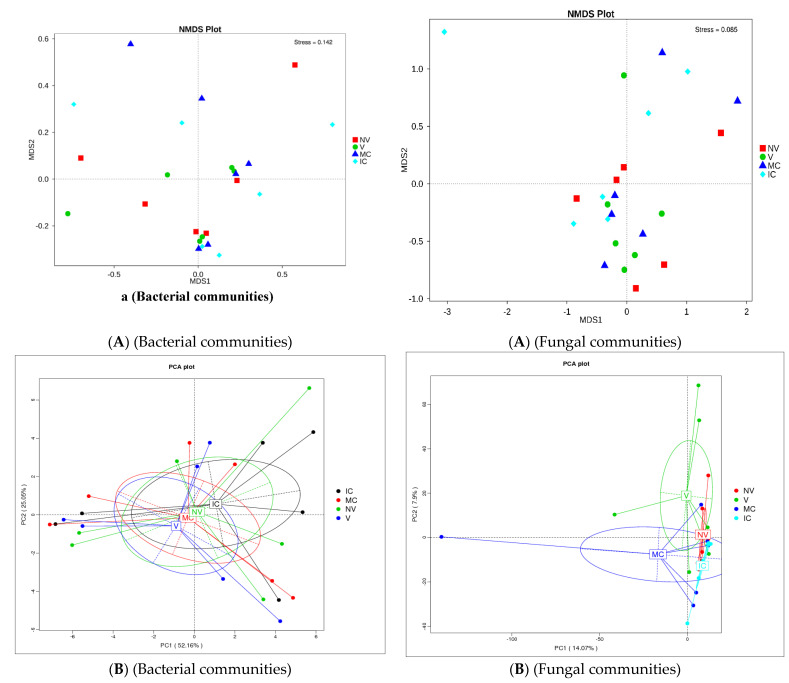
Distribution of microbial communities. (**A**) NMDS ordination of microbial community composition by cropping systems. The distance between data points reflects the extent of variation. Samples belonging to the same group are in the same color. When the value of stress factor is less than 0.2, it is considered that NMDS is reliable. (**B**) Principal coordinate analysis (PCoA) plot of bacterial and fungal community composition using Bray–Curtis distance metric at the OTU level to evaluate the similarities among the cropping system levels. Each dot represents the bacterial or fungal community composition, and the axis titles indicate the percentage variation. Where: IC, intercropping; MC, monocropping; V, vigorous; and NV, non-vigorous.

The study was conducted to reveal microbial diversity and comparison of rhizosphere microbial communities between two contrasting banana cropping systems (MC vs. IC) and monocrop vigor (NV vs. V) in Uganda and Tanzania. 16S rDNA (V3–V4) and ITS2 amplicon deep sequencing via Illumina Miseq generated approximately 1.9 and 3.9 million reads, respectively. A total of 1.8 million reads from 16S and 1.6 million reads from ITS2 were clustered into OTUs at 97% sequence identity after the removal of low-quality reads via the UCLUST algorithm. Following read quality control, 37,276 bacterial and 44,170 fungal reads were retained. A rarefaction curve for alpha diversity was drawn demonstrating that the sequencing data accounts for the whole diversity present in the samples (Supplementary Figure S2). In both bacterial and fungal community, there were changes observed in the α-diversity. The α-diversity of microbiome in bulk soil was greater than rhizosphere soil and root samples (observed species, Chao, ACE; *p* < 0.05) (Supplementary Tables S1 and S2). Additionally, differences were observed in alpha-diversity of rhizosphere microbes at the cropping systems level (Supplementary Figure S3 and Supplementary Tables S1 and S2). The cropping systems impacted the bacterial and fungal communities. MC and NV displayed high diversity of the bacterial communities. On the other hand, fungal community diversity was higher under the IC and V. Bray–Curtis distance metric and unweighted uniFrac associated with permutest and pairwise comparison was used to determine the microbial community variability (Supplementary Figure S2). No significant difference was observed in the β-diversity among the cropping systems as well as sampling compartments. In addition, difference analysis based on ANOSIM was performed that showed a minimal microbial β-diversity difference of the cropping systems and plant vigor (Supplementary Tables S3 and S4). Additionally, there was a minimal microbial β-diversity difference of the cropping systems. A common pattern was observed using principal coordinate analysis (PCoA), accounting for 52.2% and 14.1% differences in bacterial and fungal communities, respectively (Figure 3). The MC and NV were clustered far away from IC and V cropping systems, respectively. PCoA of bacterial communities was performed and it was observed that the different cropping systems were clustered together as well as grouped with the bulk, rhizosphere soil, and root samples indicating similarities in their microbial communities. On the other hand, fungal communities of IC were nested within MC. Operational taxonomy units (OTUs) count was more under NV than V cropping systems for bacterial communities. The rhizosphere soil was the major reservoir of bacterial and fungal communities in all the cropping systems. Venn diagrams and box plots on number of OTUs were produced in the UCLUST algorithm in order to compare the shared and unique OTUs number belonging to two cropping systems (NV-V and MC-IC) from bulk, rhizosphere soil, and roots (Supplementary Figure S3). It was observed that the OTUs of two cropping systems (NV-V and MC-IC) did not differ much from each other. However, there were differences in the OTUs richness of each cropping system at different locations, e.g., the number of microbial OTUs in Tanzania was less compared to Uganda, depicting that environmental conditions and plant root exudates are also key factors driving changes in the belowground bacterial and fungal community composition. With regards to locations, the samples collected from various cropping systems in Uganda (892/142 for NV-V; 860/101 for MC-IC) displayed the greatest diversity and richness in core OTUs than from Tanzania (591/71 for NV-V; 625/12 for MC-IC) for bacterial and fungal communities, respectively (Supplementary Figure S3).

### 3.4. Cropping Systems (NV-V and MC-IC) Impact on Bacterial Microbiome Structure and Composition

**Figure 4:**
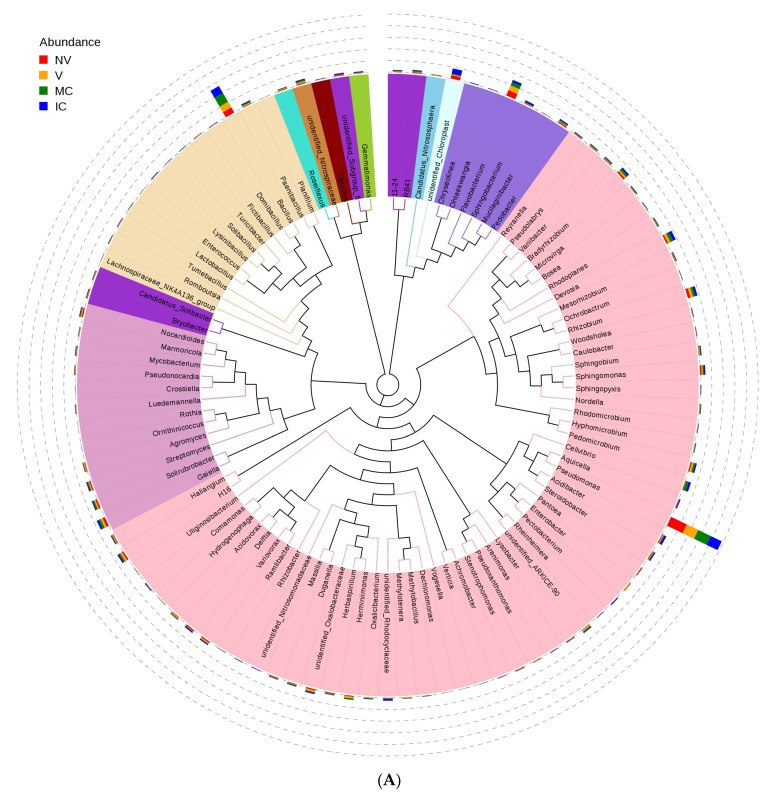

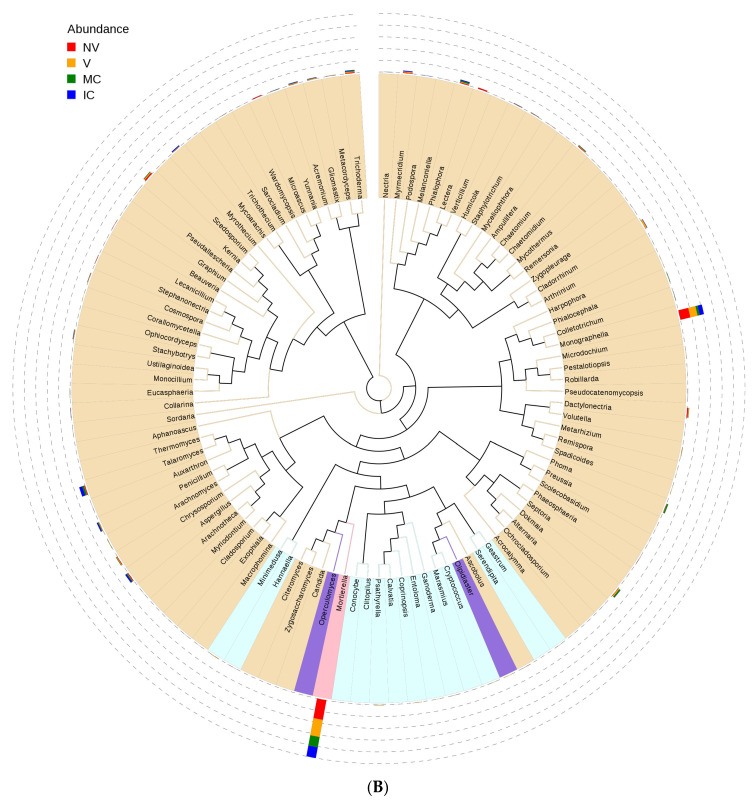
An evolutionary tree at the genus level across all the samples. Different colors of the branches represent different phyla. Relative abundance of each genus in each sample was displayed outside the circle and different colors represent different samples. (**A**) bacterial communities and (**B**) fungal communities. Where: IC, intercropping; MC, monocropping; V, vigorous; and NV, non-vigorous.

Furthermore, we studied the phylogenetic relationships of species at the genus level using multiple sequence alignments to understand the changes of specific bacterial communities. The evolutionary tree displays the relative abundance of each genus (the top 100 genera) for all the cropping systems based on the phylogenetic relationships between all OTUs representative sequences (Figure 4). Variations in microbial composition and abundance between bulk, rhizosphere soil, and roots were also observed, implying involvement of plant metabolite exchanges in driving microbiome variations. Different cropping systems (MC vs. IC) and vigor (NV vs. V) also had different microbial community structure. Both NV and V cropping systems had high abundance of phylum- Proteobacteria, Firmicutes, and Actinobacteria. However, the dominating composition of phylum- Bacteroidetes and Cyanobacteria were only found in NV cropping systems. On the other hand, both MC and IC systems had a relatively higher abundance of phylum- Proteobacteria, Firmicutes, Actinobacteria, and Bacteroidetes. However, phylum-Nitrospirae was only present in MC, unlike in IC systems which was rich in phylum- Acidobacteria and Cyanobacteria (Figure 4).

**Figure 5:**
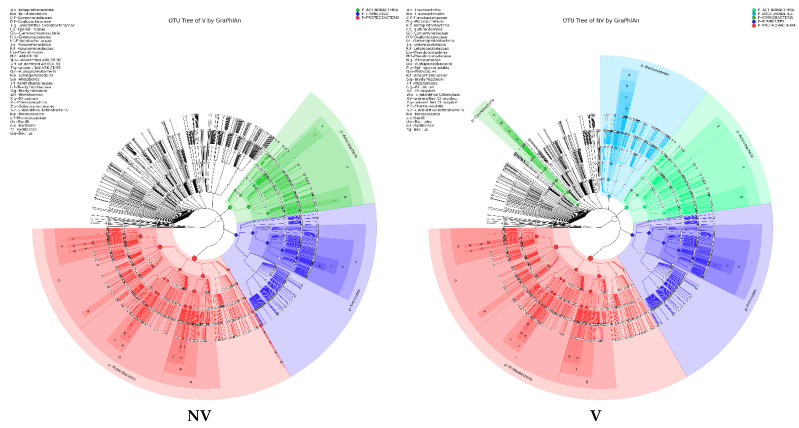

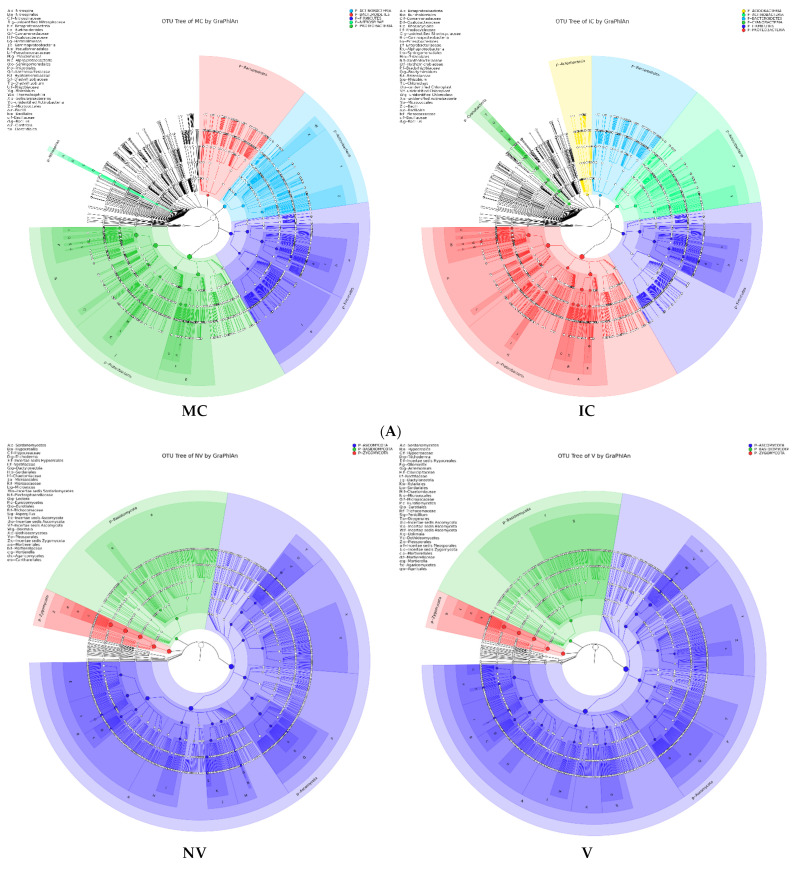

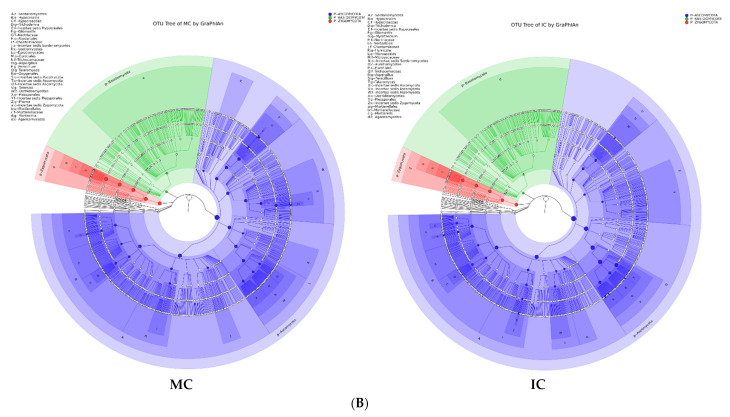
Taxonomy visualization. OTU annotation tree constructed by GraPhlAn. The size of circles stands for abundance of species. Different colors stand for different phyla. Solid circles stand for the top 40 species with high abundance. (**A**) bacterial communities and (**B**) fungal communities. Where: IC, intercropping; MC, monocropping; V, vigorous; and NV, non-vigorous.

**Figure 6:**
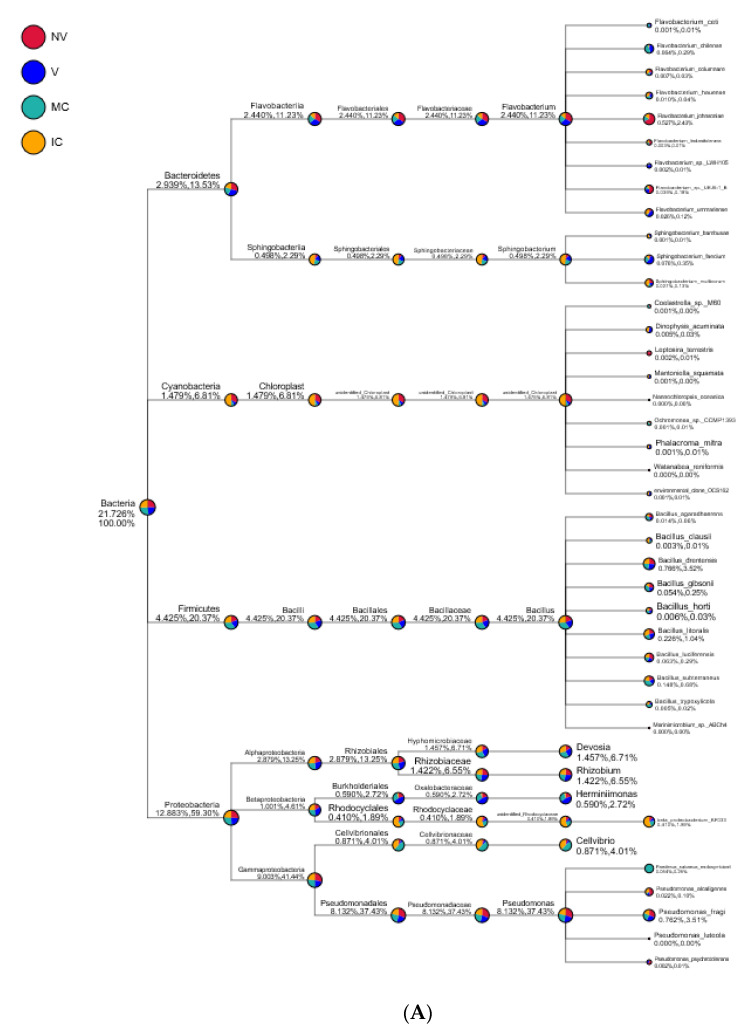

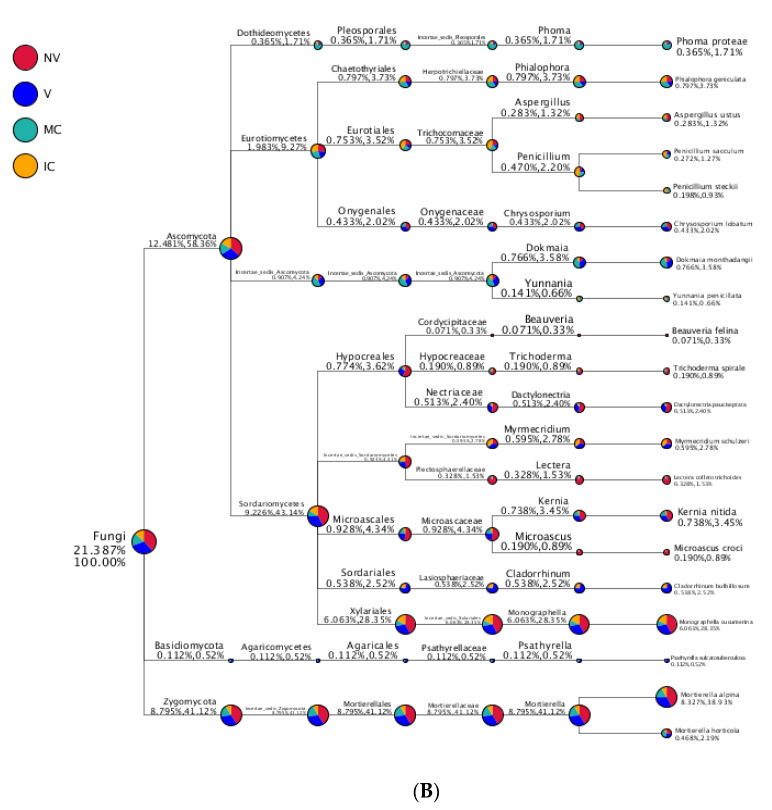
Taxonomy tree of cropping systems in plantain rhizosphere. Different colors represent different taxonomic ranks. The size of circles represents the relative abundance of species. The first number below the taxonomic name represents the percentage in the whole taxon, while the second number represents the percentage in the selected taxon. (**A**) bacterial communities and (**B**) fungal communities. Where: IC, intercropping; MC, monocropping; V, vigorous; and NV, non-vigorous.

Proteobacteria, Actinobacteria, Acidobacteria, Bacteroidetes, Firmicutes, Cyanobacteria, Thaumarchaeota, Gemmatimonadetes, Chloroflexi, and Verrucomicrobia were the 10 dominant phyla accounting for 94.95–98.49% of all bacterial taxa in the bulk soil, rhizosphere, and roots of the cropping systems (NV-V and MC-IC). Proteobacteria dominated the rest of nine phyla with a relative abundance of 12.89–59.30% in different cropping systems and became the most abundant phylum (Figure 5 and Supplementary Table S5). Proteobacteria had the same classes Alphaproteobacteria (2.88–13.25%), Betaproteobacteria (1.00–4.61%), and Gammaproteobacteria (9.00–41.44%) dominated in all the cropping systems (NV-V and MC-IC). Bacteroidetes were dominated by Flavobacteriia and Sphingobacteriia which accounted for 2.44–11.23% and 0.49–2.29% of the total taxa, respectively. The dominant families of classes Alphaproteobacteria and Gammaproteobacteria were Hyphomicrobiaceae and Pseudomonadaceae, respectively. With the presence in rhizosphere soil and roots of all the cropping systems, genus *Pseudomonas* was dominating in NV cropping systems accounting for 37.43% of the total diversity. V cropping system were dominating by *Devosia* (6.71%), *Rhizobium* (6.55%), and *Herminiimonas* (2.72%). MC systems were highly dominating by *Bacillus* (20.37%) and *Cellvibrio* (4.01%) (Figure 6). Various compartments of the different cropping systems (NV-V and MC-IC) hold these microbial communities with the relative abundance of 93.39–96.5%. However, compared to rhizosphere soil and roots, bulk soil had a relatively lower microbial abundance, illustrating root exudates and cropping systems’ importance in structuring the soil microbiome. For example, the phylum Proteobacteria was greatly confined to the rhizosphere region of MC and V compared with IC and NV cropping systems with a relatively lower abundance, respectively, indicating that banana roots and cropping systems do exert effects on bacterial phyla (Figure 6).

### 3.5. Cropping Systems (NV-V and MC-IC) Impact on Fungal Microbiome Structure and Composition

In the case of fungal taxa, Ascomycota, Basidiomycota, Zygomycota, Chytridiomycota, Glomeromycota, and Incertae_sedis were the top six dominant phyla in the different cropping systems (NV-V and MC-IC). Ascomycota dominated the rest of five phyla with a relative abundance of 12.48–58.36% in different samples and became the most abundant phylum (Figure 5 and Supplementary Table S6). Ascomycota had the same classes Dothideomycetes (0.36–1.73%), Eurotiomycetes (1.9–9.27%), Incertae_sedis (0.9–4.24%), and Sordariomycetes (9.23–43.14%) dominated in all the cropping systems (NV-V and MC-IC). Basidiomycota was dominated by Agaricomycetes which accounted only for 0.11–0.52% of the total taxa. Zygomycota was dominated by Incertae_sedis which accounted for 8.79–41.12% of the total taxa. Additionally, MC systems were observed with very high dominance of *Phoma* spp. (1.71%), whereas high abundance of *Penicillium* spp. (2.20%) was found in IC systems. *Aspergillus* spp. (1.32%) and *Trichoderma* spp. (0.89%) were the two important species confined to NV cropping systems. A high relative abundance of *Dokmaia* spp. (3.58%) were observed in V cropping systems (Figure 6).

### 3.6. KEGG Metabolic Pathways Enrichment

**Figure 7:**
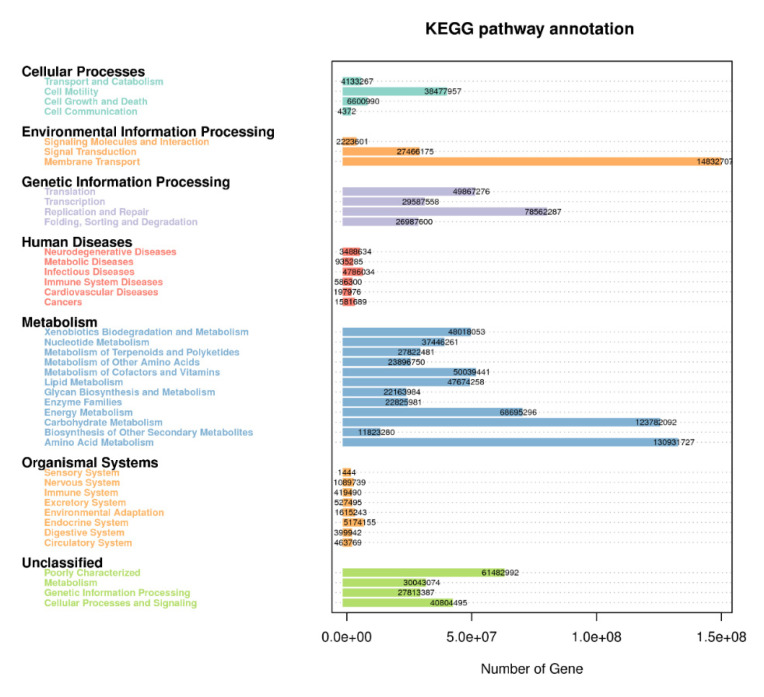

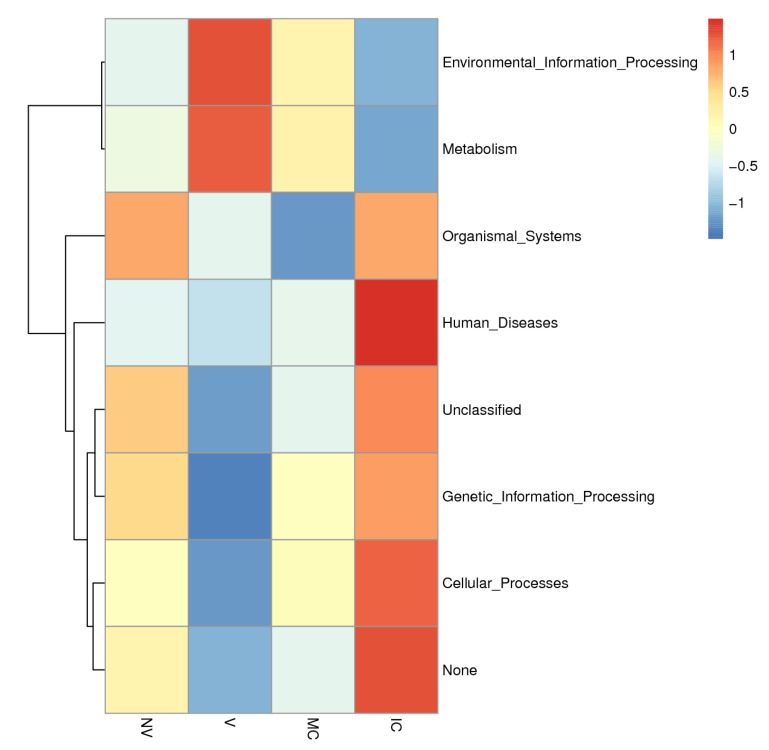
KEGG metabolic pathways enrichment in the functional microbial communities. The heatmap shows the profiles of microbial community functions based on the relative abundance of KEGG metabolic pathways (https://www.kegg.jp/kegg/kegg1.html accessed on 10 August 2021). The dendrograms show the hierarchical clustering of columns and rows respectively using Euclidean distance.

In order to further investigate the pathways of the functional diversity, microbial community functional annotations were obtained by blasting against the KEGG Orthology (KO) database. The PICRUSt analysis was applied to gain insight into the detailed metabolic and functional profiles of bacterial community which identified six level KEGG Orthology groups (KOs). These categories include cellular processes, environmental information, genetic information, metabolism, organismal system, and human diseases (Figure 7). Among the six functional categories at level one, the metabolism, environmental information processing, and genetic information processing were the dominant functional groups, accounting for 53.5%, 17.9%, and 15.8% of the total abundance of functional genes, respectively. The predicted functional profiles corresponding to level one KEGG pathways across all the samples are depicted (Figure 6), where the microbial communities in the different cropping systems (NV-V and MC-IC) possessed diverse metabolic genes involved in various mechanisms. These predominant pathways were mainly related to transport and catabolism, cell motility, cell communication, membrane transport, translation, transcription, replication and repair, folding, sorting and degradation, signal transduction, amino acid metabolism, carbohydrate metabolism, energy metabolism, enzyme families, and metabolism of lipid, nucleotide, and other compounds (Figure 7).

## 4. Discussion

### 4.1. Physico-Chemical and Enzaymatic Caharcteristics

In the present investigation, we focused our studies on the microhabitat and diverse cropping systems (NV-V and MC-IC), specific microbial communities, and their functional alliance under same management practices. First, we studied the rhizosphere soil of all the sampling locations and compared between two countries (Tanzania and Uganda) comprehensively and carefully. We examined the soil physico-chemical properties, soil nutrient contents, and enzymatic activities to know the relationship between sampling locations of the two countries. It was evident that pH of the soil has crucial impacts on crop growth and microbial community structures [29]. Compared with the rhizosphere of Tanzania, the soil of Ugandan sites was acidic (Figure 2). Both fungal and bacterial communities related to different cropping systems may appear as the pH changes in the soil. pH of soil is determined by various factors including fertilizers history, topography, mineral content, climate, and soil texture [30,31]. SOC (soil organic carbon) is another important component that determines structure of microbial communities in the soil. In comparison to the Tanzanian locations, SOC was significantly (*p* ≤ 0.05) higher in Ugandan locations. Interestingly, in our study SOC had direct influence on the microbiome diversity where Ugandan soils were observed with greatest microbial richness in terms of core OTUs compared to Tanzanian soils. SOC, a component of soil organic matter, was observed with measurable variations between locations which could be due to diverse cropping systems that were either just maintained without any inputs or in other cases by adding additional crop residues in the fields over a period of time. Although the results showed no significant difference between Tanzanian and Ugandan soils for most nutrients, all the studied soil nutrients were comparatively different for both countries with their high requirements in the soil (Figure 2). For instance, nitrogen, phosphorus, and potassium are the essential component of nuclear and membrane structure in plants. These essential nutrients regulate metabolism, stimulate root and shoot growth, and enhance the tolerance against biotic and abiotic stress conditions [32]. Soil enzymes determine the fertility index, health, and quality of the soil, and thus insinuate to the microbial community structures. Four soil enzymes (phosphatase, invertase, urease, and catalase) were measured in this study. The outcomes showed that the activity of invertase and catalase in Tanzania soil were significantly lower than those in the soil of Uganda. Furthermore, there was no critical contrast of urease and phosphatase activities observed in the soil of different locations (Figure 2). The ubiquitous soil enzyme invertase breaks down sucrose into fructose and glucose providing carbon for soil microorganisms’ growth, which could be another obvious reason for higher microbial diversity and richness in Ugandan soils. More importantly, in Ugandan soils, the decomposition and transformation of peroxides in soil can be possible by the soil enzyme catalase which helps in diminishing antagonistic effects of peroxides on soil quality [33]. Urease plays out the hydrolysis of urea to carbon dioxide and ammonia, which imparts a crucial role in the soil nitrogen cycling [34]. Phosphatase helps the crops through the retention and usage of natural phosphorus present in the soil and can be a fundamental marker for the hydrolysis of phosphorus compounds.

### 4.2. Cropping Systems Richeness and Diversity

Alongside with the morphological changes, cropping systems results in the variations in root exudates compositions which leads to successional shifts in microbial diversity in the root vicinity. This suggests that different cropping systems prefer peculiar microbial communities having their own functional potential [35,36]. Richness estimators (Ace and Chao 1) and diversity indices (Shannon and Simpson) analysis revealed a richer microbial community in MC and V than that of IC and NV, respectively. The conceivable explanation is the progressions in the profile of plant root exudates composition and general association with roots as compared to bulk and rhizosphere soil. The interaction among plant species richness, land-use history, and soil types could affect microbial community structure [37]. In all the cropping systems (NV-V and MC-IC), the dominant bacterial taxonomic groups include Proteobacteria, Firmicutes, and Actinobacteria. These phyla have been characterized as the most common soil inhabitants [38,39]. The bacterial community structures were also significantly different between the different cropping systems. The relative abundances of the dominant bacterial phyla (Proteobacteria, Firmicutes, and Actinobacteria) were greater in NV and V; the relative abundances of the dominant bacterial phyla (Bacteroidetes and Cyanobacteria) were only identified in NV. The significant presence of Nitrospirae were observed in the MC systems compared to richer IC systems with high abundance of Acidobacteria and Cyanobacteria (Figure 5A).

Moreover, cropping systems physiology, inconsistency of root exudates, and their interactions with microbes are crucial determinants of soil diversity at genus level [40]. The endosphere (roots) of bananas was identified as a phenomenal microhabitat for *Pseudomonas* in the NV cropping systems (Figure 6). *Pseudomonas* strains are the notable plant colonizers responsible for the beneficial plant-microbe interaction through growth enhancement, stress attrition, and antagonism for phytopathogens [41,42]. Additionally, increased relative abundances of *Devosia* and *Rhizobium* in V cropping systems explained that a nonlegume crop such as banana is a strong competitor for soil inorganic nitrogen, and thus N_2_ fixation capacity in mono banana crop may be further enhanced by inter or mixed cropping systems [43,44]. Furthermore, MC systems were observed with very high abundance of *Bacillus* and *Cellvibrio* spp. and identified as the main component in the many disease-suppressive soils [45]. Additionally, the well-known plant colonizers, growth promoters and nitrogen fixers, decreased abundance of *Bacillus* and *Cellvibrio* spp. in IC systems, which might be due to the presence of highly diverse and antagonistic microbial communities. Apart from the abiotic parameters of plants, their microhabitats also differ in the microbial communities [46,47] as the roots exudes extremely salubrious low molecular weight compounds into the rhizosphere region of plants [48,49], responsible for retaining the most potent microbiomes. In contrast, the bulk and rhizosphere region of the different cropping systems harbor specifically adapted and diverse microbial communities [50]. Furthermore, on all the farm locations, these traditional cropping systems were managed by one family over many generations, and bananas are cultivated by the vegetative suckers from old banana mats/corms. As a result, banana corm serves as the storage house and transportation mode of endophytic microorganisms of the de novo shoots in successive cropping systems (NV-V and MC-IC). For bananas, we obtained higher OTU’s in the rhizosphere than in the roots and bulk soil of the cropping systems, in general. Notwithstanding, we noticed the rhizosphere area served as a pleasant environment for highly abundant and diverse microbial communities’ structure, which could straightforwardly affect the different cropping systems [51,52]. Additionally, several macronutrients including nitrogen, phosphorus, and potassium add a vital role in the traditional smallholder cropping systems of SSA. The process of nitrogen fixation and solubilization of phosphate and potassium were also influenced by the structure of bacterial taxa in soils, which is very important for soil quality and thus cropping systems behavior in the region.

Several unexpected findings including the unique microbiology in the banana rhizosphere and the dominant role of Enterobacteriaceae were investigated as a major component in the bacterial communities of all the different cropping systems (NV, V, and IC) except MC (Figure 6). The high abundance of Enterobacteriaceae can be explained by amendments in the cropping systems of both human and animal manure in these traditional farms. This is often the only source of nutrients to the crops in the farms managed by smallholder farmers in SSA. Several antibiotic resistance genes and plasmids in bacteria were investigated that were isolated from the soil treated with animal manures [53]. Indeed, many enterics were observed as the positive colonizers in the rhizosphere regions of the plant tissues [54]. However, we need further tests to assess their pathogenic capacity.

In the case of fungal groups, Ascomycota, Basidiomycota, and Zygomycota dominated all the cropping systems (NV-V and MC-IC). Incertae_sedis was the dominant fungal group found in the roots of all the cropping systems (NV-V and MC-IC). Members of Mortierellaceae were identified as the major component of fungal communities in rhizosphere and bulk soil of all the cropping systems. These outcomes showed that plant species changed the predominant fungal phyla in soil.

### 4.3. Metabolic Pathways Revealed in Cropping Systems

To provide the structural and functional linkages of microbial communities, various studies have focused on the evolutionary history, metabolic pathways, and gene regulation of microbiomes [55,56]. We used the PICRUSt tool to fill the gap between soil microbial communities and different cropping system (NV-V and MC-IC) practices in SSA by symbolizing the metabolic and functional capabilities of the identified microbiome across a broad range of samples [57,58]. We inferred three major functional categories corresponding to the metabolism, environmental information processing, and genetic information processing on KEGG pathways. In our study, functional genes explained the relative abundance of microbial communities and their involvement in major biological processes (Figure 7). Additionally, in all the cropping systems, the major proportion of bacterial communities were represented by Proteobacteria commonly responsible for biological carbon and nitrogen cycling process in soil ecosystems [59]. Other heterotrophic bacteria, belonging to Actinobacteria and Proteobacteria, were also known to degrade amino acids, sugars, and organic acids [60,61]. We additionally observed critical effects on the cropping systems practices on KEGG pathways related with different microbial communities. These outcomes reinforce the future work on how different cropping systems impacts the functional properties of the soil microbiome.

Moreover, there are emerging threats to banana cropping systems and ecosystem health such as recent diseases including *Fusarium* wilt caused by soil borne fungal pathogen in bananas [62,63]. Balanced plant-microbe (indigenous) interactions and upgrading soil systems with diverse group of beneficial microbial treatments (single or consortia) are the promising keys to avoid such pathogenic invasions. Our study showed that smallholder cropping systems in SSA are the hopeful territories for essential microbial communities which requires very much overseen nature-based choice and their restrained introduction to stabilize agricultural ecosystems.

## 5. Conclusions

In the present study, we found that different smallholder cropping systems significantly differ the composition and functional profile of microbial communities. Significant variations and disorders were observed on the overall α and β-diversity of inhabiting microbial communities in different cropping systems. Although MC and NV are more constructive in beneficial microbial community structures, IC and V cropping systems could also promote some functional bacterial communities, suggesting their complex relationships and more species dependence. Therefore, shifting the different cropping systems, for instance from MC to IC, change the microbial diversity, and the microbial community in turn manipulates various biological processes favorable for soil health, crop productivity, and environmental sustainability. These findings provide deep insights into the role of cropping systems in shaping microbial communities, their functions, and provide directions in identifying the best cropping model in agroecosystems.

## Figures and Tables

**Table 1:** Geographical and metrological parameters of sampling locations.

S. No.	Parameters	Tanzania			Uganda		
		Meru	Rombo	Rungwe	Fort Portal	Luweero	Kyenjojo
1.	Sampling time	May 2019	May 2019	May 2019	June 2019	June 2019	June 2019
2.	Average annual temperature (°C)	Max. 25.8 ± 0.5 Min. 16.3 ± 0.7	Max. 23.5 ± 0.5 Min. 14.6 ± 0.9	Max. 21.6 ± 0.7 Min. 12.8 ± 0.8	Max. 25.0 ± 0.5 Min. 15.2 ± 0.7	Max. 26.8 ± 0.6 Min. 19.2 ± 0.7	Max. 25.8 ± 0.7 Min. 15.9 ± 0.5
3.	Soil temperature at the time of sampling (°C)	21 ± 0.5	17.7 ± 0.5	16.4 ± 0.5	20.2 ± 0.5	23.4 ± 0.5	21.9 ± 0.5
4.	Average annual precipitation (mm)	233.3	110.3	955.0	160.5	125.3	158.5

## Data Availability

Sequence reads were submitted to NCBI under Accession No. PRJNA630414.

## Supplementary Materials

The following supporting information can be downloaded at: https://www.mdpi.com/article/10.3390/biology11050756/s1, Table S1: Alpha diversity at countries and cropping systems level (16S). Where: IC, intercropping; MC, monocropping; V, vigorous; NV, non-vigorous; Table S2: Alpha diversity at countries and cropping systems level (ITS). Where: IC, intercropping; MC, monocropping; V, vigorous; NV, non-vigorous; Table S3: β-diversity (ANOSIM) at countries and cropping systems level (16S). Where: IC, intercropping; MC, monocropping; V, vigorous; NV, non-vigorous; Table S4: β-diversity (ANOSIM) at countries and cropping systems level (ITS). Where: IC, intercropping; MC, monocropping; V, vigorous; NV, non-vigorous; Table S5: Top 10 dominant phyla of bacterial taxa Where: IC, intercropping; MC, monocropping; V, vigorous; NV, non-vigorous; Table S6: Top 10 dominant phyla of fungal taxa Where: IC, intercropping; MC, monocropping; V, vigorous; NV, non-vigorous; Figure S1: Cropping systems in sub-Saharan Africa (SSA). a, monocropping (MC) b, intercropping (IC) c, vigorous (V) d, non-vigorous (NV); Figure S2: Alpha diversity curves of bacterial (above) and fungal (below) communities indicating the biodiversity of the samples. (a) Rarefaction curves (b) Rank abundance curves. Where: IC, intercropping; MC, monocropping; V, vigorous; NV, non-vigorous; BS, bulk soil; RS, rhizosphere soil; RT, roots; T, Tanzania and U, Uganda; Figure S3: Venn diagram and box plots on number of OTUs for the bacterial and fungal communities of the cropping systems. The observed OTUs for each treatment were produced in the UCLUST algorithm to show the shared and unique OTUs. Only the most abundant OTUs among all the samples were represented. Where: IC, intercropping; MC, monocropping; V, vigorous; NV, non-vigorous; BS, bulk soil; RS, rhizosphere soil; RT, roots; T, Tanzania and U, Uganda.
