# Supplementary material for: Compositional Shifts in Microbial Diversity under Traditional Banana Cropping Systems of Sub-Saharan Africa
Source: Biology (Basel). 2022 May 16;11(5):756. doi: 10.3390/biology11050756 (PMC9138362; doi:10.3390/biology11050756)
Supplement: Supplementary file 1 [file biology-11-00756-s001.zip › biology-1649814-supplementary.pdf]

Table S1. Alpha diversity at countries and cropping systems level (16S). Where: IC, intercropping; MC, monocropping; V, vigorous; NV, non-vigorous

Group	diff	lwr	upr	p adj
MC-IC	-0.02483333	-2.207223	2.157556	0.9999880
NV-IC	0.11166667	-2.070723	2.294056	0.9989156
V-IC	0.07166667	-2.110723	2.254056	0.9997118
NV-MC	0.13650000	-2.045889	2.318889	0.9980279
V-MC	0.09650000	-2.085889	2.278889	0.9992986
V-NV	-0.04000000	-2.222389	2.142389	0.9999498

Table S2. Alpha diversity at countries and cropping systems level (ITS). Where: IC, intercropping; MC, monocropping; V, vigorous; NV, non-vigorous

Group	diff	lwr	upr	p adj
MC-IC	0.54216667	-2.219047	3.303381	0.9455827
NV-IC	-0.04516667	-2.806381	2.716047	0.9999643
V-IC	1.08133333	-1.679881	3.842547	0.6958938
NV-MC	-0.58733333	-3.348547	2.173881	0.9322970
V-MC	0.53916667	-2.222047	3.300381	0.9464071
V-NV	1.12650000	-1.634714	3.887714	0.6687700

Table S3. β-diversity (ANOSIM) at countries and cropping systems level (16S). Where: IC, intercropping; MC, monocropping; V, vigorous; NV, non-vigorous

Group	R-value	P-value
MC-NV	-0.0963	0.799
V-NV	-0.08148	0.78
V-MC	-0.1148	0.855
IC-NV	-0.07778	0.693
IC-MC	-0.09815	0.827
IC-V	-0.1093	0.856

Table S4. β-diversity (ANOSIM) at countries and cropping systems level (ITS). Where: IC, intercropping; MC, monocropping; V, vigorous; NV, non-vigorous

Group	R-value	P-value
MC-V	-0.01481	0.524
IC-V	-0.01667	0.44
IC-MC	-0.0537	0.693
NV-V	-0.1444	0.975
NV-MC	-0.113	0.831
NV-IC	-0.08056	0.843

Table S5. Top 10 dominant phyla of bacterial taxa Where: IC, intercropping; MC, monocropping; V, vigorous; NV, non-vigorous

Taxonomy	Proteobacteria	Actinobacteria	Firmicutes	Acidobacteria	Bacteroidetes	Cyanobacteria	Thaumarchaeota	Gemmatimonadetes	Chloroflexi	Verrucomicrobia
NV	0.446137	0.182462	0.064673	0.09523	0.064065	0.019984	0.016555	0.032707	0.026551	0.014458
V	0.444278	0.201614	0.064378	0.08062	0.059071	0.006277	0.046303	0.024052	0.025809	0.015482
MC	0.420324	0.178219	0.109067	0.08712	0.057296	0.005231	0.042342	0.025183	0.02449	0.017158
IC	0.471316	0.153572	0.075984	0.090205	0.066877	0.029922	0.016863	0.027924	0.019134	0.014288

Table S6. Top 10 dominant phyla of fungal taxa Where: IC, intercropping; MC, monocropping; V, vigorous; NV, non-vigorous

Taxonomy	Sordariomycetes	Incertae_sedis _Zygomycota	Eurotiomycetes	Agaricomycetes	Dothideomycetes	Pezizomycetes	Incertae_sedis _Ascomycota	Leotio mycetes	Chytridio mycetes	Tremello mycetes
NV	0.465025	0.227521	0.069868	0.052629	0.03845	0.003675	0.007629	0.004369	0.000396	0.001992
V	0.380243	0.220341	0.052089	0.028746	0.041853	0.008682	0.017393	0.002566	0.003369	0.000939
MC	0.518129	0.141716	0.066321	0.031014	0.062197	0.021672	0.018797	0.006697	0.00083	0.002332
IC	0.37456	0.10888	0.084156	0.026573	0.031206	0.002068	0.00684	0.00575	0.000208	0.000253

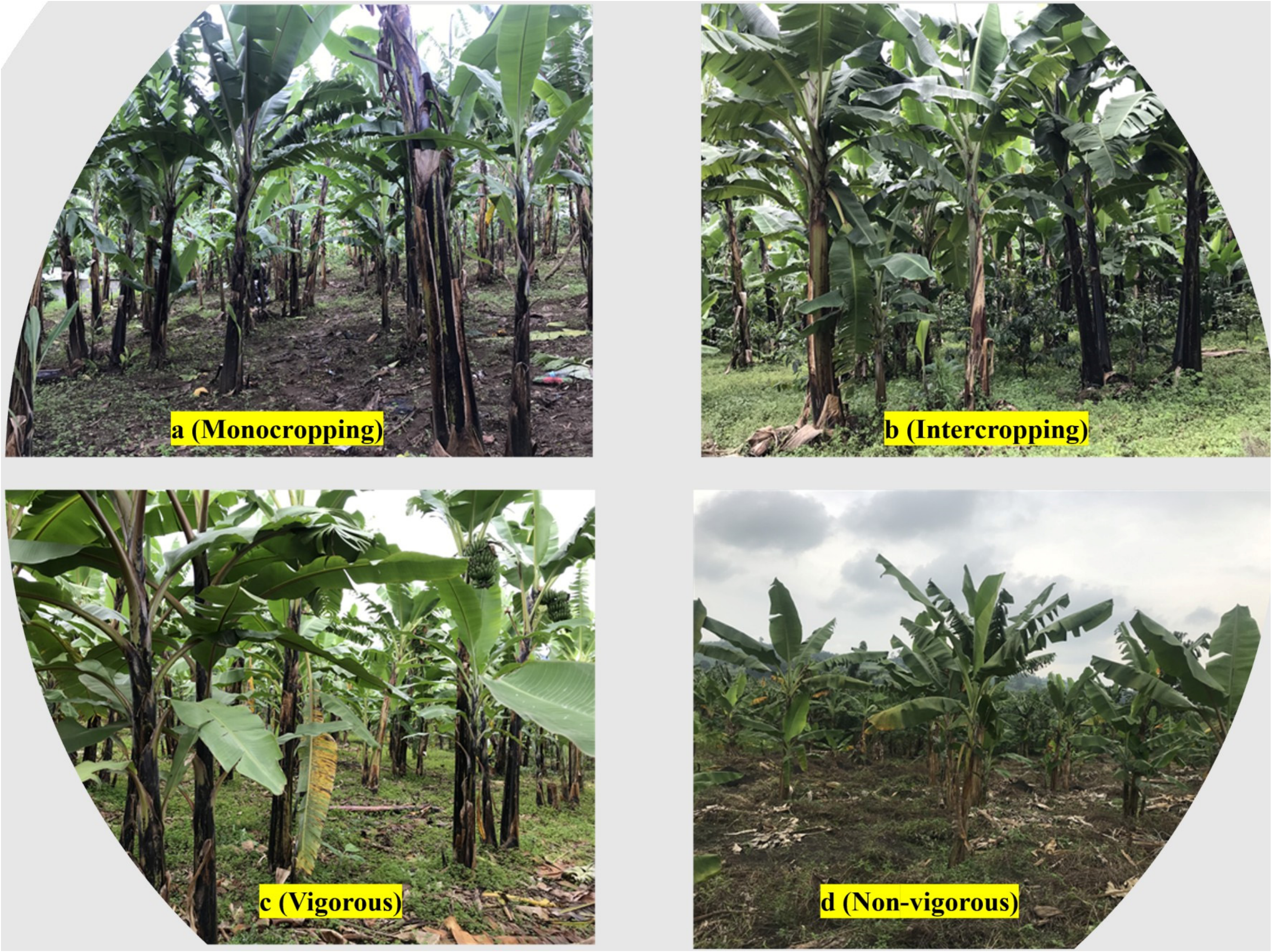

Figure S1. Cropping systems in sub-Saharan Africa (SSA). a, monocropping (MC) b, intercropping (IC) c, vigorous (V) d, non-vigorous (NV)

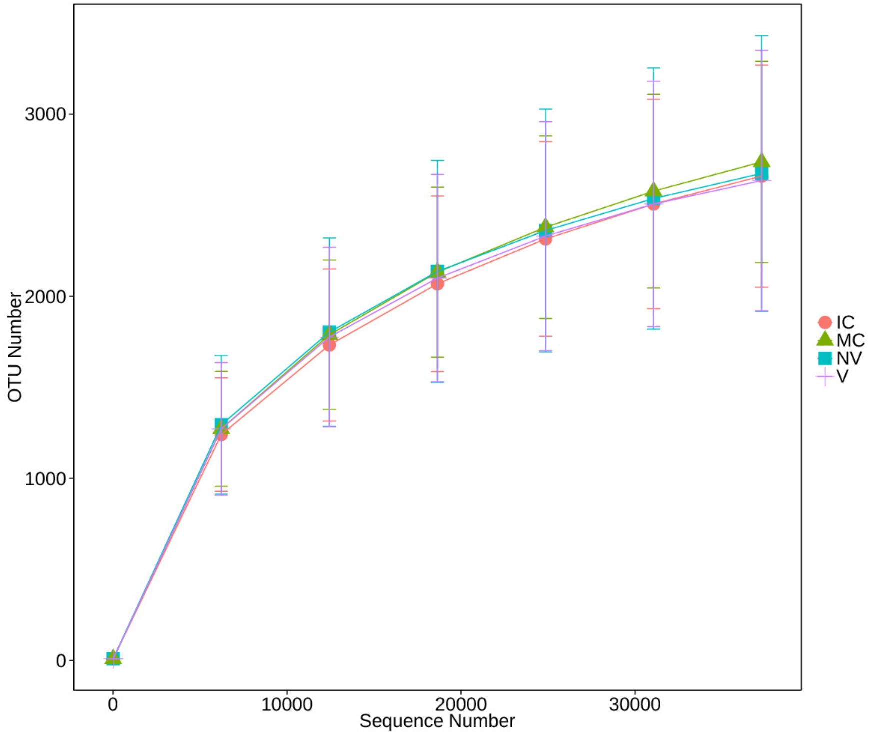

(a- bacterial)

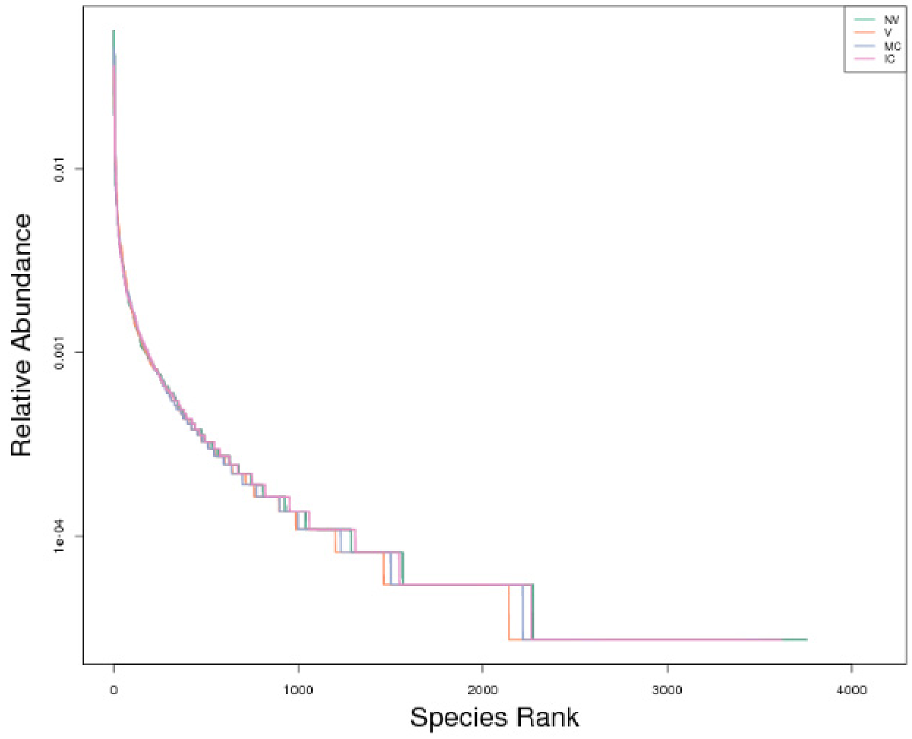

(b- bacterial)

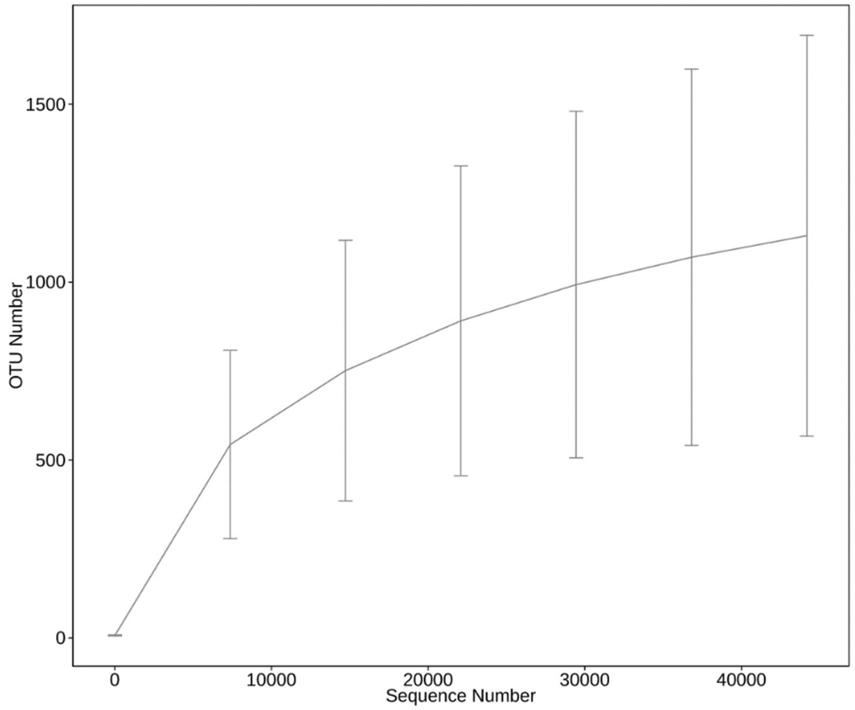

(a- fungal)

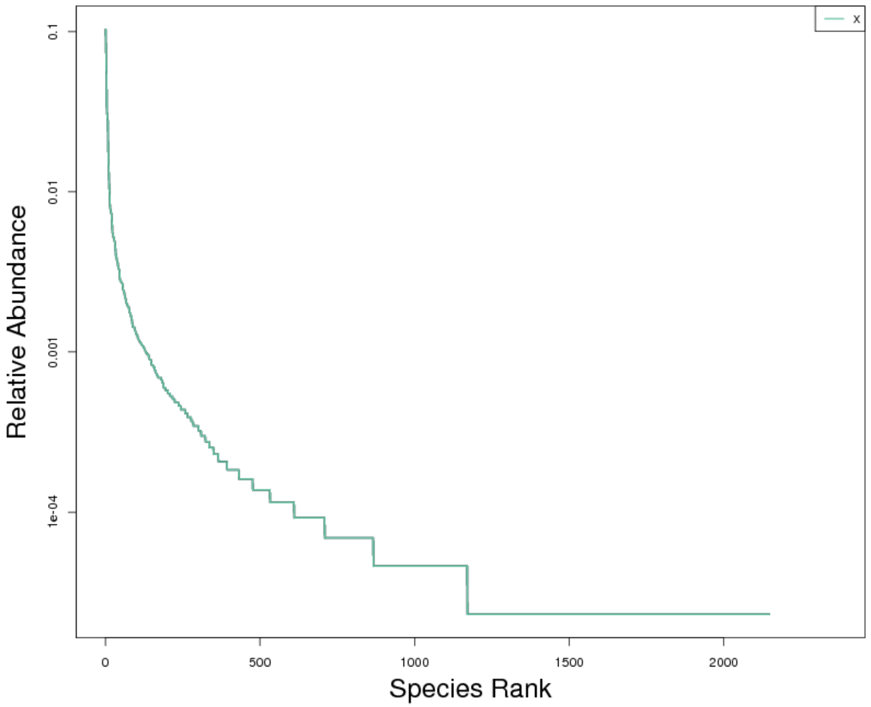

(b- fungal)

Figure S2. Alpha diversity curves of bacterial (above) and fungal (below) communities indicating the biodiversity of the samples. (a) Rarefaction curves (b) Rank abundance curves. Where: IC, intercropping; MC, monocropping; V, vigorous; NV, non-vigorous; BS, bulk soil; RS, rhizosphere soil; RT, roots; T, Tanzania and U, Uganda

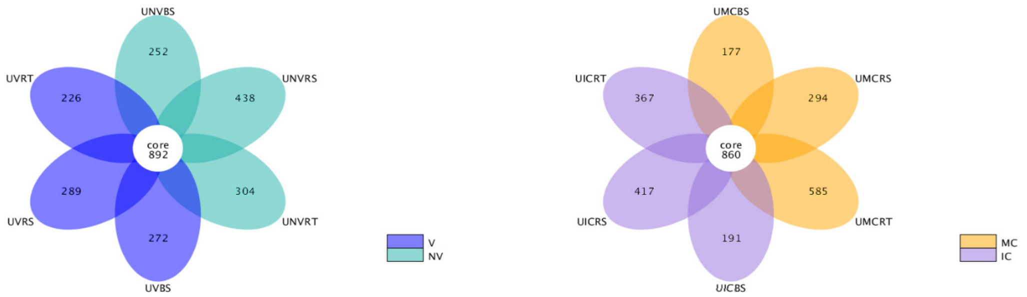

Bacterial communities in Uganda

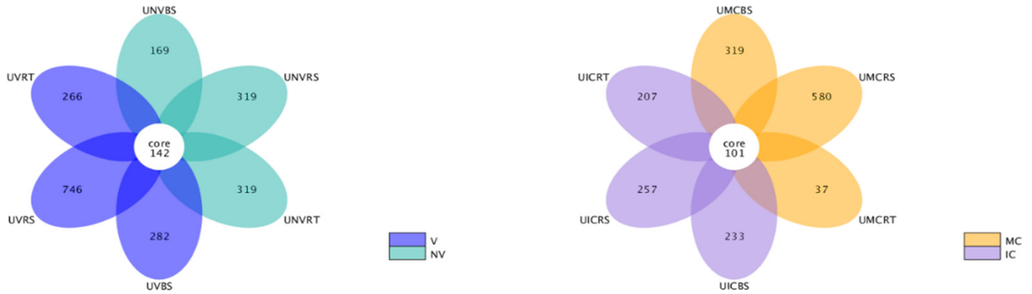

Fungal communities in Uganda

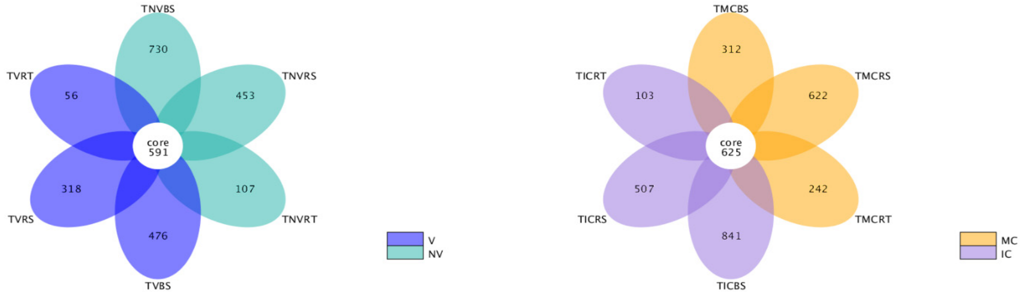

Bacterial communities in Tanzania

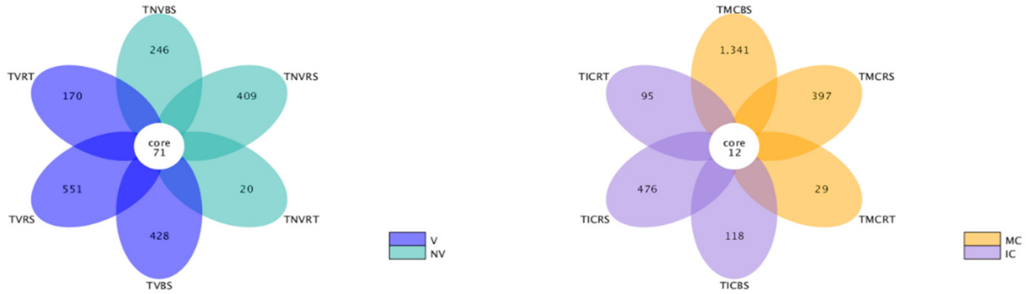

Fungal communities in Tanzania

Figure S3. Venn diagram and box plots on number of OTUs for the bacterial and fungal communities of the cropping systems. The observed OTUs for each treatment were produced in the UCLUST algorithm to show the shared and unique OTUs. Only the most abundant OTUs among all the samples were represented. Where: IC, intercropping; MC, monocropping; V, vigorous; NV, non-vigorous; BS, bulk soil; RS, rhizosphere soil; RT, roots; T, Tanzania and U, Uganda
